# Availability of postabortion care services in Ethiopia: Estimates from a 2020 national sample of public facilities

**DOI:** 10.1016/j.conx.2022.100087

**Published:** 2022-10-28

**Authors:** Hayley V. McMahon, Celia Karp, Suzanne O. Bell, Solomon Shiferaw, Assefa Seme, Mahari Yihdego, Linnea A. Zimmerman

**Affiliations:** a Department of Health, Behavior and Society, Johns Hopkins Bloomberg School of Public Health, Baltimore, MD, United States; b Department of Population, Family and Reproductive Health, Johns Hopkins Bloomberg School of Public Health, Baltimore, Maryland, United States; c School of Public Health, Addis Ababa University, Addis Ababa, Ethiopia

## Abstract

**Objectives:**

Unsafe abortion is a leading cause of global maternal mortality and morbidity. This study sought to estimate availability of essential postabortion care (PAC) services among publicly managed health facilities in Ethiopia.

**Study design:**

Data from public hospitals and health centers in Ethiopia were collected in 2020. Among facilities offering labor and delivery, we assessed the proportion that: (1) offered PAC, (2) were equipped for each PAC signal function, and (3) were equipped for all PAC signal functions falling within their scope of care by facility type.

**Analysis:**

Our primary outcome was PAC service provision status. Descriptive statistics summarized the proportion of hospitals and health centers, respectively, categorized as each PAC status and with necessary equipment for individual signal functions. Per Federal Ministry of Health (FMOH) guidelines, hospitals are expected to provide comprehensive PAC, while health centers are expected to provide basic PAC.

**Results:**

Altogether, 69.1% (*n* = 94) of hospitals were equipped to provide comprehensive PAC, and 65.2% (*n* = 131) of health centers were equipped for basic PAC. Least available signal functions included obstetric surgery among hospitals (83.8%; *n* = 114) and uterine evacuation among health centers (84.6%; *n* = 170).

**Conclusion:**

Meaningful progress has been made toward achieving the Ethiopian FMOH's goal of universal PAC service availability at hospitals and health centers by 2020. Despite this, sizable gaps remain and may endanger maternal health in Ethiopia, underscoring a need for continued prioritization of PAC services.

**Implications:**

Ethiopia's commitment to PAC has fostered a service landscape that is stronger than many other low-resource settings; however, notable shortcomings are present. Further research is needed to understand the potential role of clinical training and supply-side interventions.

## 1 Introduction

When performed according to medical standards, abortion rarely results in complications [1]. However, injury and infection caused by abortions that are induced through unsafe means continue to significantly contribute to maternal morbidity and mortality in areas where it is illegal or difficult to access safe abortion services [2]. While widespread access to safe abortion care is the most effective primary prevention for mortality related to unsafe abortion, the provision of postabortion care (PAC) services to treat complications of unsafe abortion is essential secondary prevention [3]. PAC is a lifesaving component of emergency obstetric care that the World Health Organization (WHO) defines with five key signal functions: removing retained products of conception, administering parenteral antibiotics, administering uterotonic drugs, transfusing blood, and performing obstetric surgery [4]. Deaths resulting from unsafe abortion are largely preventable through PAC, yet unsafe abortion remains a top cause of global maternal mortality [5]. The magnitude of unsafe abortion is of particular concern in East Africa, where more than three-quarters (76.1%) of all abortions that take place in the region are unsafe [6].

Ethiopia revised its abortion law in 2005 to include circumstances beyond cases of life or health endangerment for the pregnant person. With this new policy, Ethiopia expanded its definition of legal abortion to permit the termination of pregnancies that involve a poor fetal prognosis, result from rape or incest, or are carried by a minor or disabled person [7]. Although these changes have increased access to safe abortion, nearly half of abortions in the country still occur outside health facilities [8]. While abortion can be safely self-managed outside of the formal healthcare system, many self-induced abortions in sub-Saharan Africa are still carried out through unsafe means, such as inserting herbs, sharp objects, or caustic substances into the vagina. Complications resulting from these dangerous methods can require emergency PAC services to prevent permanent disability or death [6,9,10,11].

The Ethiopian Federal Ministry of Health (FMOH) reports that complications resulting from unsafe abortions are the fifth leading cause of hospital admission for female patients nationally [12]. In 2010 alone, researchers found that 52,607 women received treatment for induced abortion complications at Ethiopian health facilities. More than 40% of these patients were severely ill when they sought care and required urgent intervention [13]. Worse still, the majority of those in need of urgent PAC do not receive it. Estimates from 2014 indicate that only one-quarter of those with postabortion complications in Ethiopia accessed health services [14].

The availability of high-quality PAC services in health facilities plays a significant role in the ability of pregnant people to access these critical services during obstetric emergencies. To address this threat to public health, the FMOH identified the following Performance Target to be achieved by 2020 in its 2016 National Reproductive Health Strategy: “Hundred percent of health centers and hospitals provide comprehensive abortion care services as per the law and per the guidelines” [15]. These FMOH guidelines specify that health centers are expected to provide basic PAC services for minor complications, and hospitals are expected to provide more advanced, comprehensive PAC services for major complications [15]. In recent years, progress has been made to bolster health facility capacity to provide PAC services across Ethiopia. While these developments are promising, no estimates have been generated on the status of PAC services availability in Ethiopia since 2014 [16,17]. Research is needed to gage success and guide strategic interventions to improve access to PAC services and meet FMOH targets. This study aims to fill this gap by estimating the extent to which publicly managed health facilities in Ethiopia report providing PAC services and are equipped to do so — 15 years after the liberalization of the country's abortion law.

## 2 Materials and methods

### 2.1 Data

This analysis uses data from Performance Monitoring Action Ethiopia (PMA Ethiopia), a collaborative study between Addis Ababa University (AAU), FMOH, and Johns Hopkins Bloomberg School of Public Health (JHSPH). PMA Ethiopia generates cross-sectional and longitudinal data on reproductive health indicators among women and the health facilities in their communities to inform national and regional government priorities and policies. We use data collected from health facilities as part of PMA Ethiopia's Service Delivery Point (SDP) survey, which includes a range of measures on reproductive health services, facility infrastructure, referral capabilities, and medication and equipment stocks [18]. Enumeration areas (EAs) were selected through a multistage sampling approach, with region and residence as strata. Once EAs were selected, local health officials were consulted to identify facilities assigned to provide health services to the EA. All public facilities serving an EA were selected. All private facilities in the kebele, the lowest level administrative unit in Ethiopia, were listed, and up to three were randomly selected.

Data collection supervisors in Ethiopia administered in-person surveys to facility officials from October to December 2020. Given the context of COVID-19, data collection included enhanced safety protocols, such as mandatory masking and social distancing. Facility staff provided oral consent to participate. The survey response rate was 97.0%. Institutional review boards at AAU and JHSPH provided ethical approval for PMA Ethiopia and deemed the facility survey exempt as nonhuman subjects research. Details about the sampling and survey protocol used for PMA Ethiopia are available in Zimmerman et al. [18].

### 2.2 Measures

Based on the methodology developed by Healy and colleagues, we adapted the WHO's four PAC signal functions — removing retained POC, administering parenteral antibiotics, transfusing blood, and performing obstetric surgery — to define indicators of PAC service availability and map them to SDP survey items [4,19]. Per WHO's clinical guidance for managing incomplete abortion, we distinguished the removal of retained products of conception (POC) as two signal functions: uterine evacuation and uterotonics [20]. Although WHO does not explicitly include immediate postabortion contraception or intravenous (IV) fluids as signal functions for PAC, they are commonly found as PAC signal functions within the existing literature, so we chose to include them in our analysis [16,19,21]. Table 1 details our seven adapted signal functions, their clinical indications, and the survey items used to operationalize them.

**Table 1:** Signal function indicators used to determine the minimum acceptable resources needed to provide basic and comprehensive postabortion care (PAC), adapted from WHO guidance [4,20]

Signal function (SF)	Clinical indication	Item from PMA Ethiopia SDP survey
BASIC PAC		
SF1: Uterine evacuation	Hemorrhage	“For each item I list, please indicate if it is available, and if available, please show me the item: manual vacuum aspirator (MVA) and cannula.”
SF2: Administration of uterotonics	Sepsis; hemorrhage	“For each item I list, please indicate if the item is available, and if available, please show me the item: injectable oxytocin; misoprostol tablet (600 mg; not in combined form).”
SF3: Administration of parenteral antibiotics	Sepsis; intra-abdominal injury	“For each item I list, please indicate if the item is available, and if available, please show me the item: injectable ampicillin; injectable gentamicin.”
SF4: Provision of postabortion family planning	Prevention	“Is immediate postpartum family planning provided at this facility?”
COMPREHENSIVE PAC (BASIC +)		
SF5: Performance of obstetric surgery	Intra-abdominal injury	“Is major obstetric surgery (e.g., cesarean, hysterectomy) provided at this facility?”
		“How many health workers with the following qualifications work in this facility: Emergency surgery and obstetrics office (M.*Sc*. level)?”
SF6: Performance of a blood transfusion	Hemorrhage; Intra-abdominal injury	“Is blood transfusion provided at this facility?”
SF7: Administration of intravenous (IV) fluids	Hemorrhage	“For each item I list, please indicate if the item is available, and if available, please show me the item: Intravenous solution: either Ringer's lactate, D5NS, or NS infusion.”

Signal functions (SF) were assessed as available for each facility using a dichotomous measure: all essential supplies available (coded as one) or at least one essential resource unavailable (coded as zero). Supplies were required to be in-stock and observed on the day of the survey to be considered available. As seen in Table 1, facilities met the criteria for SF1 (uterine evacuation) if they had a manual vacuum aspirator (MVA) kit. For SF2 (uterotonics), facilities needed to have either misoprostol tablets or injectable oxytocin available. SF3 (antibiotics) was assessed as the availability of injectable ampicillin or injectable gentamicin, and SF4 (IV fluids) required facilities to have at least one of three IV fluids: Ringer's lactate, 5% dextrose in sodium chloride (D5NS), or normal saline (NS) [20]. SF5-SF7 were measured through direct report from the facility administrator [18]. If a response to the relevant survey item was not provided, we assumed the signal function was not present.

We used the dichotomous measures of signal function availability to create a composite, categorical variable for assessing our primary outcome: facility PAC service provision status. We categorized facilities into one of four groups based on signal function availability: *No PAC Offered, Does Not Meet Minimum Basic PAC Criteria, Equipped for Basic PAC*, and *Equipped for Comprehensive PAC* (Table 2). Facilities categorized as *Basic PAC* had all necessary supplies for SF1-SF4 but were missing SF5, SF6, or SF7. Facilities categorized as *Comprehensive PAC* met all the criteria for SF1−SF7. For example, if a facility reported providing PAC services but did not have parenteral antibiotics (SF3) for the treatment of sepsis, it was categorized as *Does Not Meet Minimum Basic PAC Criteria.* Comprehensive PAC was not calculated for health centers as it does not fall within their scope of practice [15]. Table 2 defines our four categories of PAC service provision status. Additionally, average monthly PAC caseloads were computed from facility-reported register data, including inpatient and outpatient care for postabortion services.

**Table 2:** Variable definitions to categorize facilities according to PAC provision status

PAC service provision status	Definition
*No PAC Offered*	This facility reported that it does not provide PAC services.
*Does Not Meet Minimum Basic PAC Criteria*	This facility reported that it provides PAC services but does not have the minimum signal functions required to provide basic PAC services.
*Equipped for Basic PAC*	This facility reported that it provides PAC services and has all of the minimum signal functions required to provide basic PAC services. It does not have the minimum signal functions required to provide comprehensive PAC services
*Equipped for Comprehensive PAC*	This facility reported that it provides PAC services and has all of the minimum signal functions necessary for PAC at all levels.

### 2.3 Sample

A total of 735 facilities (hospitals, health centers, health clinics, health posts, pharmacies, and drug shops/rural drug vendors) across 10 regions completed the PMA Ethiopia SDP survey; data were not collected in Tigray due to ongoing conflict in the region. We first restricted the sample to hospitals and health centers (*n* = 359), which are designated by FMOH guidance as facilities expected to provide PAC services. Per the survey design, questions about PAC supplies, services, and commodities were only asked to facilities offering labor and delivery (L&D) services, thereby limiting our sample to facilities with L&D services in their scope (*n* = 347). We further restricted our sample to include only government-managed hospitals and health centers to maintain focus on alignment with FMOH guidance, which resulted in a final analytic sample of 337 facilities, including 136 hospitals and 201 health centers.

### 2.4 Analysis

We used descriptive statistics to describe facility characteristics and calculate the proportion of hospitals and health centers that (1) reported offering PAC services, (2) were equipped for each PAC signal function, and (3) were equipped for all PAC signal functions falling within their scope of care (basic/comprehensive) by facility type. Next, average monthly PAC caseloads were estimated to assess the magnitude of PAC clients receiving care according to each PAC provision status and explore differences in PAC provision status based on client volumes. Analyses were conducted in Stata 17 [22].

## 3 Results

### 3.1 Facility characteristics

Table 3 describes the characteristics of the 337 facilities analyzed. Health centers comprised more than half (59.6%; *n* = 201) of the sample, and most (93.8%; *n* = 316) facilities overall reported providing PAC services. Facilities were concentrated across three regions: Oromia (24.6%; *n* = 83), Amhara (24.4%; *n* = 80), and Southern Nations, Nationalities, and People's Region (SNNPR) (23.2%; *n* = 78). Average monthly PAC caseloads varied widely across regions, ranging from 3.3 PAC clients in Gambella to 21.1 in Harari, and by facility type, ranging from an average of 2.1 PAC clients in health centers to 27.6 in hospitals.

**Table 3:** Characteristics of public health facilities offering labor and delivery (*N* = 337)

	Total facilities(*N* = 337)% (n)	Monthly PAC caseloadsmean (sd)
Facility type		
Health centers	59.6 (201)	2.1 (3.12)
Hospitals	40.4 (136)	27.6 (61.7)
Reports providing PAC services		
Yes	93.8 (316)	13.2 (42.4)
No	6.2 (21)	–
Region		
Addis Ababa	9.5 (32)	8.6 (14.7)
Afar	4.5 (15)	4.2 (5.5)
Amhara	24.4 (80)	11.3 (18.3)
Benishangul-Gumuz	3.0 (10)	6.4 (11.3)
Dire Dawa	3.0 (10)	4.7 (8.9)
Gambella	3.3 (11)	3.3 (7.4)
Harari	2.1 (7)	21.1 (29.7)
Oromia	24.6 (83)	13.4 (18.7)
SNNPR	23.2 (78)	18.4 (81.3)
Somali	3.3 (11)	9.0 (17.2)
Tigray	–	–

*Notes: Column totals presented. Data unavailable in Tigray due to lack of data collection during conflict.

### 3.2 Signal functions

**Fig. 1:**
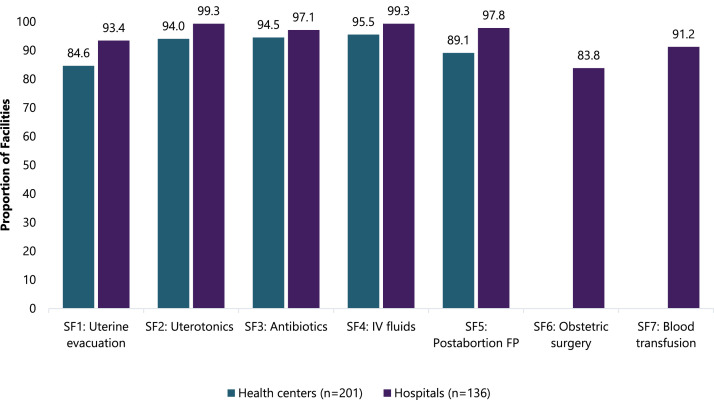
Proportion of public health centers and hospitals in Ethiopia (*N* = 337) meeting the minimum criteria to provide each PAC signal function, by facility type, 2020.

Fig. 1 shows the proportion of health centers and hospitals equipped for each signal function. Hospitals were generally well-equipped to provide all signal functions. Among hospitals, obstetric surgery was the least available (83.8%; *n* = 114) signal function required for comprehensive PAC services. Among health centers, the signal function with the least available equipment was uterine evacuation (84.6%; *n* = 170), followed by postabortion family planning (89.1%; *n* = 179). Obstetric surgery and blood transfusions were not calculated for health centers as these comprehensive signal functions do not fall within their scope of care.

### 3.3 PAC service provision status

**Fig. 2:**
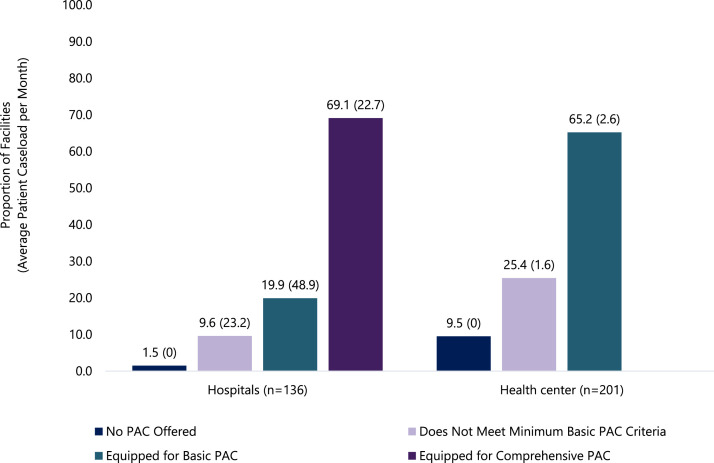
PAC service provision status of public hospitals and health centers in Ethiopia and average monthly PAC patient caseload by PAC provision status (*N* = 377), 2020.

**Fig. 3:**
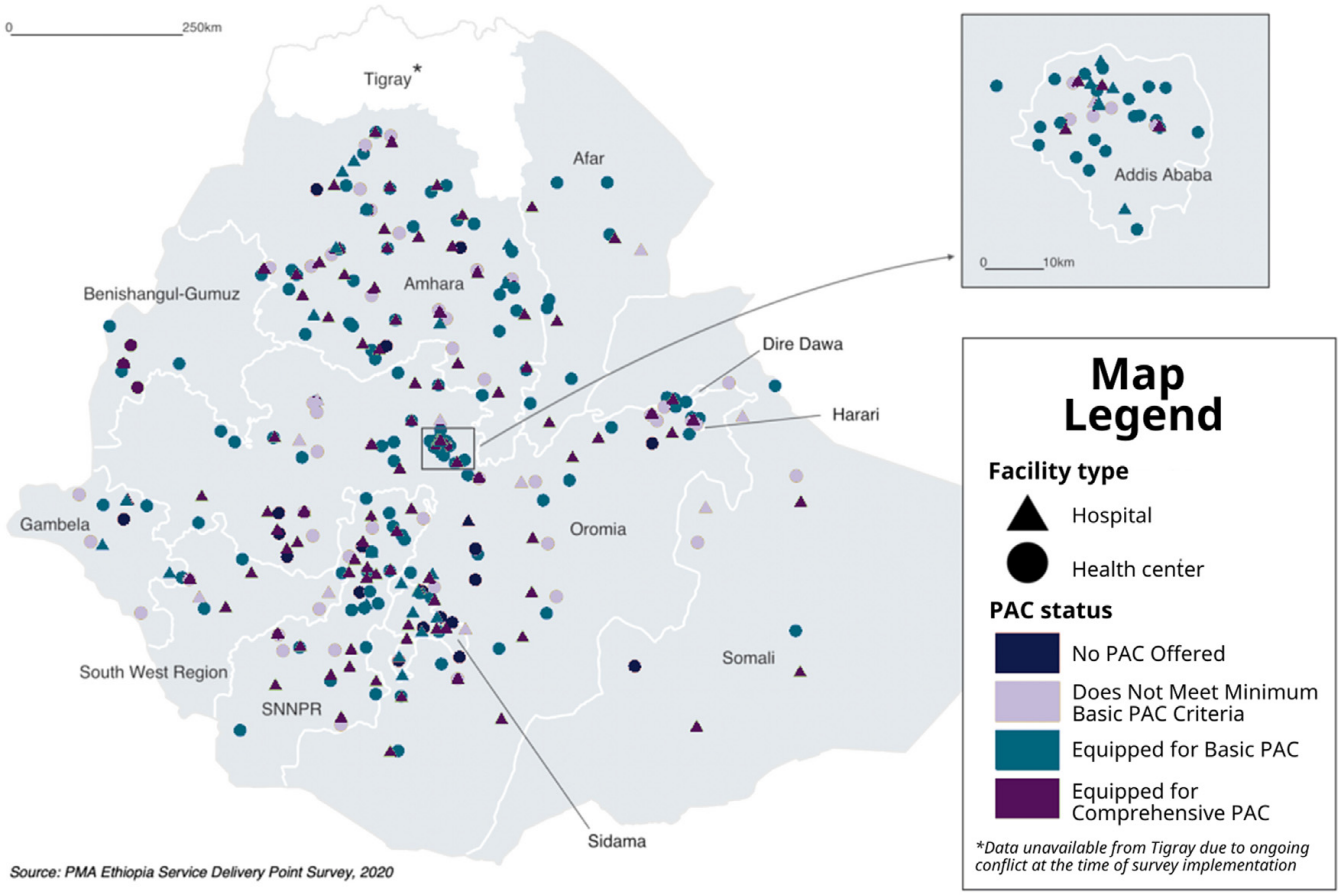
Distribution and PAC service provision status of public hospitals and health centers in Ethiopia (*N* = 377), 2020.

Fig. 2 presents PAC provision status by facility type, and Fig. 3 illustrates the geographic distribution of the categorized facilities. Overall, seven out of 10 hospitals (69.1%) had the necessary equipment for all comprehensive PAC signal functions. Approximately one in five hospitals (19.9%) were only equipped to provide basic PAC and 9.6% did not meet the minimum criteria even for basic PAC. Among health centers, roughly two-thirds (65.2%) were equipped for basic PAC. In comparison, 10.0% of health centers reported not providing PAC, and, while one-quarter reported providing PAC, the facilities were not equipped to meet the minimum criteria for basic care. Among health centers that provided PAC, the average monthly caseload ranged from 1.6 patients for those characterized as not meeting minimum basic PAC criteria to 2.6 patients for those that were equipped to provide basic PAC. Among hospitals, this ranged from 23.2 patients among facilities not equipped to provide basic PAC to 48.9 patients for those equipped to deliver basic PAC*.*

## 4 Discussion

This study estimated the extent to which publicly managed health facilities in Ethiopia report providing PAC services and are equipped to deliver them. Findings indicate significant progress in the development of PAC services in Ethiopia, particularly when contrasted with recent estimates from similar low-resource settings; however, substantial gaps in service coverage remain [3,21].

The vast majority of facilities in our sample reported providing PAC services. Hospitals were generally well-equipped for individual PAC signal functions, with most were equipped to provide care for all signal functions. While service availability was still relatively high for obstetric surgery (83.8%), it was the least available signal function among hospitals, highlighting a clear opportunity for strengthening availability and readiness of essential services.

Despite seeing many strong indicators of success, such as 95.5% of health centers being equipped to provide IV fluids, we also identified several deficiencies among facilities that warrant attention. Approximately 15% were not equipped for uterine evacuation, and more than 10% were not equipped to provide postabortion family planning. These signal functions are fundamental components of basic PAC services for the prevention and treatment of common obstetric emergencies and subsequent unintended pregnancies [23,24]. These gaps must be prioritized to reduce morbidity and mortality.

Per the Performance Targets from the FMOH's National Reproductive Health Strategy, by 2020 all health centers should be providing basic PAC services, and all hospitals should be providing comprehensive PAC services by 2020 [15]. Meaningful progress is being made toward achieving this critical goal, with roughly 65%–70% of facilities providing designated care. Despite this, further work is needed to ensure health centers and hospitals are equipped to deliver essential reproductive health care and protect women's health and well-being.

Our study also identified a surprising number of facilities—including one-quarter of health centers and 10% of hospitals—that reported providing PAC but lacked the minimum signal functions required to do so. While these facilities were underequipped to deliver care according to minimum clinical standards, these facilities still reported average monthly caseloads of 1.6 patients for health centers and 23.2 for hospitals, raising concern about the quality of care provided. Of similar concern, we found the highest average monthly patient caseload among hospitals that were only equipped for basic PAC services. Additional research is needed to investigate the cause of the discrepancies between standards, reporting, and practice to improve the delivery and availability of care.

Results must also be interpreted in light of limitations. First, contextual factors related to the COVID-19 pandemic and conflict in Ethiopia's northern region may have influenced results by disrupting supply chains or diverting the focus of clinical resources. Additionally, the sample of health facilities identified in PMA Ethiopia is not nationally representative; the sampling strategy overrepresents hospitals and public facilities. To avoid biased estimates, we stratified by health center and hospital. We also excluded private facilities due to an insufficient number in the sample (*n* = 10), which means these findings are not generalizable to facilities run by nonprofits or religious organizations. Survey items used to ascertain PAC provision status were only asked of facilities offering labor and delivery services, thereby excluding PAC services that may be available at public health centers and hospitals that do not offer labor and delivery care, though these facilities were a small minority (*n* = 12). In addition, the survey did not require interviewers to visually confirm supplies related to family planning, obstetric surgery, or blood transfusion; these measures were self-reported by facility staff and could have resulted in an overestimate. Finally, these findings do not refer to the accessibility or quality of PAC services. Facilities being equipped does not directly translate into care if patients cannot reach them or properly trained clinicians are not available to provide care.

Additional research is needed to explore the quality of the PAC services being provided and the role that clinical training and supply-side interventions could play in strengthening these health systems. PAC also serves additional purposes beyond emergency obstetric care for complications as general follow-up care for both self-managed and clinician-provided abortion, and this should be further explored in terms of how PAC services can improve the overall quality and patient-centeredness of abortion care [25,26]. Future research should examine inequities in geographic access to both safe abortion care and postabortion care services to better identify opportunities for improving reproductive health services for all.

## 5 Conclusion

Our analysis illustrates progress toward the FMOH Performance Target of universal PAC service provision at hospitals and health centers by 2020; however, concerning gaps in PAC service availability at multiple facility levels still persist [15]. Inequities in structural capability require continued supply chain and service improvement efforts to increase availability of PAC services across health facilities in Ethiopia. This research offers an updated perspective of what has been achieved for PAC provision in the context of the FMOH Performance Target set in 2016, outlines actionable information about the extent to which PAC services are available in the country, and identifies targets to guide strategic clinical interventions.
